# Cardiorespiratory fitness and accelerometer-determined physical activity following one year of free-living high-intensity interval training and moderate-intensity continuous training: a randomized trial

**DOI:** 10.1186/s12966-020-00933-8

**Published:** 2020-02-26

**Authors:** M. E. Jung, S. R. Locke, J. E. Bourne, M. R. Beauchamp, T. Lee, J. Singer, M. MacPherson, J. Barry, C. Jones, J. P. Little

**Affiliations:** 1 School of Health and Exercise Sciences Faculty of Health and Social Development, The University of British Columbia Okanagan, FHSD 3333 University Way ART360, Kelowna, BC V1V 1V7 Canada; 2 The University of Bristol, Bristol, UK; 3 The University of British Columbia, Vancouver Campus, Vancouver, Canada

**Keywords:** High-intensity interval training, Physical activity adherence, Health behaviour change, Cardiorespiratory fitness

## Abstract

**Background:**

Free-living adherence to high-intensity interval training (HIIT) has not been adequately tested. This randomized trial examined changes in cardiorespiratory fitness (CRF) and accelerometer-measured purposeful physical activity over 12 months of free-living HIIT versus moderate-intensity continuous training (MICT).

**Methods:**

Ninety-nine previously low-active participants with overweight/obesity were randomly assigned to HIIT (*n* = 47) or MICT (*n* = 52). Both interventions were combined with evidence-based behaviour change counselling consisting of 7 sessions over 2 weeks. Individuals in HIIT were prescribed 10 X 1-min interval-based exercise 3 times per week (totalling 75 min) whereas individuals in MICT were prescribed 150 min of steady-state exercise per week (50 mins 3 times per week). Using a maximal cycling test to exhaustion with expired gas analyses, CRF was assessed at baseline and after 6 and 12 months of free-living exercise. Moderate-to-vigorous physical activity of 10+ minutes (MVPA10+) was assessed by 7-day accelerometry at baseline, 3, 6, 9, and 12 months. Intention to treat analyses were conducted using linear mixed models.

**Results:**

CRF was improved over the 12 months relative to baseline in both HIIT (+ 0.15 l/min, 95% CI 0.08 to 0.23) and MICT (+ 0.11 l/min, 95% CI 0.05 to 0.18). Both groups improved 12-month MVPA10+ above baseline (HIIT: + 36 min/week, 95% CI 17 to 54; MICT: + 69 min/week, 95% CI 49 to 89) with the increase being greater (by 33 min, 95% CI 6 to 60) in MICT (between group difference, *P* = 0.018).

**Conclusion:**

Despite being prescribed twice as many minutes of exercise and accumulating significantly more purposeful exercise, CRF improvements were similar across 12 months of free-living HIIT and MICT in previously low-active individuals with overweight/obesity.

## Introduction

High-intensity interval training (HIIT) has attracted substantial attention as an efficacious and time-efficient exercise strategy. Systematic reviews and meta-analyses of supervised exercise training studies consistently demonstrate HIIT improves cardiorespiratory fitness (CRF), often in a manner superior to traditional moderate-intensity continuous training (MICT) [1, 2]. HIIT also improves other aspects of cardiometabolic health and is touted to be particularly efficacious for improving fat loss [3], insulin sensitivity [4], and reducing diabetes risk [5]. However, limited research has examined adherence to HIIT outside of a laboratory setting. Initial research has demonstrated previously low-active individuals can perform HIIT while supervised in a fitness facility setting [6, 7] or in supervised group-based exercise classes in a community park [8], but studies have failed to assess free-living, independent physical activity behaviour and have been of limited duration (10–12 weeks). It is unclear whether HIIT is a truly viable exercise option, as compared to standard care MICT, for improving CRF and cardiometabolic risk in the real-world.

Self-management of a behaviour as complex as sustaining exercise over time is challenging. Exercise adherence rates are notoriously low in the general population [9, 10] and in individuals following a supervised training intervention [11, 12]. Utilizing evidence-informed behaviour change techniques is strongly encouraged for promoting exercise adherence [13]. Self-efficacy, individuals’ confidence in their skills or abilities to perform a given action, is a psychological resource necessary for the successful initiation and maintenance of health behaviors [14]. Task (i.e., confidence to perform a particular exercise modality) and self-regulatory (i.e., confidence to self-manage, self-monitor exercise) forms of efficacy are both important beliefs for individuals to develop throughout a behaviour change intervention to promote adherence.

Assuming previously low-active individuals will adhere to HIIT simply because it is efficacious and time-efficient is naïve and not in line with best practices of exercise self-management. With this in mind, we previously conducted a pilot study whereby 2 weeks of supervised HIIT was partnered with a brief (7 session) behaviour change counselling intervention that demonstrated preliminary evidence that previously low-active individuals with overweight/obesity at elevated risk for type 2 diabetes can adhere to HIIT over one [15] and six [16] months in free-living conditions.

The primary aim of this randomized trial was to compare differences in CRF between those performing self-selected free-living HIIT and MICT measured 6- and 12-months after a brief two-week supervised intervention combined with behaviour change counselling. Secondary aims were to examine accelerometer-measured purposeful exercise [moderate-to-vigorous physical activity in bouts of ≥10 min (MVPA10+)], body composition, and self-efficacy.

## Method

The protocol of this registered trial (ClinicalTrials.gov # NCT02164474) is published elsewhere [17]. As such, an overview of the methods is presented. CONSORT checklist provided in Additional file 1.

### Study design

*Small Steps for Big Changes* was a two-arm parallel group randomized trial that compared change in CRF and adherence to HIIT versus MICT 12 months following a two-week brief exercise counselling program in individuals who were low active and with overweight and obesity.

### Participants

Eligible participants were between the ages of 30 and 65, were low-active (i.e., engaged in 2 or less bouts of moderate and/or vigorous aerobic exercise per week in the previous 6-months), had a body mass index (BMI) between 25 and 40 kg/m^2^, and were cleared to engage in vigorous exercise using the Physical Activity Readiness Questionnaire-Plus (PAR-Q+) [18]. Participants were recruited through paper and online ad postings in the community (e.g., posters in community centres, coffee shops, online advertisements).

### Procedure

This study received clinical research ethics approval from the first author’s university research ethics board and met the ethical standards of the Declaration of Helsinki. Eligible participants provided written informed consent. An external statistician computer-generated (SAS PROC PLAN) random allocations to condition (1:1, HIIT or MICT) using permutated blocks of random size, stratified for sex; these were accessed by the project coordinator via password-protected website. Participants in both conditions completed 10 exercise sessions over a two-week period, seven of which were one-on-one supervised sessions conducted in the laboratory (exercise training plus counselling), while three were conducted at home to foster independence.

#### Exercise protocol

The exercise prescriptions for each condition were progressive and matched for estimated external work. HIIT involved sessions progressing from 4 to 10 × 1-min high-intensity intervals at ~ 80–90% VO_2_peak interspersed with 1-min rest periods at ~ 40% VO_2_peak and with 5 min of warm up and cool down. MICT involved sessions progressing from 20 to 50 min of continuous moderate-intensity exercise at ~ 45–55% VO_2_peak. All participants were exposed to a variety of exercise formats (e.g., stationary cycling, treadmill, elliptical, walking outside) and were able to self-select the exercise modality for four of the supervised sessions with the remaining three performed as stationary cycling to ensure accurate intensity based on the baseline VO_2_peak test. Participants wore a heart-rate monitor that provided them with feedback to understand the physiological exercise sensations (i.e., breathing, heart rate) associated with their prescribed exercise intensity zone. Following the two-week training program, participants were recommended to exercise three times a week performing either 10 × 1 min high intensity intervals or 50 min of continuous moderate intensity exercise. Participants could vary the number of intervals or duration to achieve of the prescribed total volume (i.e., 30 high intensity intervals or 150 moderate minutes).

#### Exercise counselling

Participants in both conditions received the same brief exercise counselling intervention delivered throughout the two-week supervised training program. Counselling was delivered in a one-on-one format at each of the seven supervised sessions (~ 10 min per session, 70 min total) and via take-home worksheets for the three home-based sessions. A detailed description of the behaviour change techniques used to promote exercise self-management are reported elsewhere [19]. Briefly, task self-efficacy to perform HIIT or MICT was primarily bolstered through: providing instruction on how to perform the behaviour, behavioural practice, and helping participants identify physiological cues associated with the assigned exercise intensity. Self-regulatory efficacy was bolstered through: providing participants with opportunities to practice, with feedback, on self-monitoring, planning, and solving exercise barriers for independent exercise. Finally, salience of the positive psychological and physiological outcomes associated with exercise engagement (i.e., outcome expectations) was fostered through education and by bringing awareness to participants’ own subjective experiences of exercise and the experiences of similar individuals.

Participants were provided with a self-monitoring mobile application [20] to track their exercise during the 12-month trial and were sent monthly booster messages through this app to reinforce the psychological mechanisms addressed in counselling sessions. Exercise trainers monitored their participants through the app and contacted them when they failed to login for three consecutive days [21].

### Measures

#### Demographic information

Age, sex, ethnicity, annual household income, marital status, and education level were collected at baseline.

#### Cardiorespiratory fitness

was measured using peak oxygen uptake (absolute and relative VO_2peak_) and peak power output (W_peak_) at baseline, 6 and 12 months. VO_2peak_ was assessed by a continuous incremental ramp (15 W/min) maximal exercise test on an electronically braked cycle ergometer (Lode Excalibur, The Netherlands) with expired gas collection (Parvomedics TrueOne 2400, Salt Lake City, Utah, USA). The metabolic cart was calibrated with a 3.0 L syringe and gases of known concentration before every test. VO_2_peak is defined as the highest 30-s average for VO_2_ (in l/min and ml/kg/min) and Wpeak the highest power achieved. Criteria for determining VO_2_ peak was indicated by a leveling (< 0.100 L・min-1) or decrease in VO_2_ with increasing workload; a plateau in heart rate (< 5 bpm) and (or) attainment of age predicted maximum heart rate; a respiratory exchange ratio > 1.1; and volitional fatigue.

#### Accelerometer-measured purposeful moderate-to-vigorous physical activity (MVPA)

adherence was assessed by accelerometry (Actigraph GT3X-BT, Actigraph, Pensacola, Florida, USA) at baseline and 3-, 6-, 9- and 12-month follow-up, using Freedson’s (1998) uniaxial cut-points [22]. In line with suggestion by Migueles’ et al. (2017) review of accelerometry cut-points [23], the driving factors for selecting Freedson (1998) cut-points was comparability between studies, as it is the most commonly used cut-points with 5-s epochs summed as counts per minute. Participants were asked to wear the accelerometer on their right hip for seven consecutive days. A total of ≥10 h of valid wear time per day was required to be included in the analyses.

Due to the intermittent nature of HIIT and the lack of standardized methods to quantify HIIT based on accelerometry, purposeful physical activity was operationalized as minutes spent in MVPA in bouts of ≥10 min (MVPA10+) [24]. MVPA10+ was also selected a priori as a measure of interest, as at the onset of the trial, bouts of 10 min or more of moderate and vigorous physical activity was considered the minimal amount required to elicit beneficial physiological adaptations [25]. In line with Watson [26], scoring the MVPA10+ variable allows for drops of up to 1-min in intensity, which means the intermittent nature of HIIT would still be captured. “Rest” periods were performed at ~ 40% VO2peak, which would not result in a drop in accelerometer counts sufficient to cause a bout of HIIT to fail to be counted, as verified in our pilot research [16].

Total minutes of moderate and vigorous physical activity was also collected and analyzed ing Actilife v.6.11. Freedson cut points were used to identify time spent in each exercise intensity [22]. We also examined the *proportion of MVPA prescription achieved*, by taking the total time spent in MVPA10+ and divided it by the number of minutes that was prescribed to participants in each condition as an indicant of meeting the prescribed exercise volume (HIIT was prescribed 75 min and MICT 150 min).

#### App-based self-monitored exercise

Participants used a mobile application [20] to self-monitor their exercise engagement. Participant could report the details of their exercise, including whether they did HIIT or MICT. Using this data as a measure of adherence to prescribed exercise, we extracted the total number of times that participants reported performing HIIT or MICT for those who self-monitored throughout the 12-month follow-up. We created weekly averages (# of bouts per week) for the first and second half of the 12-month follow-up.

#### Anthropometrics

Height and weight (SECA, 700 SECA, Hamburg, Germany), and waist circumference (WC, measured at the level of the umbilicus) [27] were taken by the same trained research assistant at each timepoint.

#### Body composition

Dual energy X-ray absorptiometry (DXA; Hologic Discovery A) scans were used to assess total body fat percentage.

#### Self-efficacy

Based on Bandura’s (2006) methodological procedures [28], task self-efficacy assessed individuals’ confidence in their abilities to perform HIIT or MICT, dependent on assigned condition. Self-regulatory efficacy assessed individuals’ confidence in their abilities to manage their exercise behaviour (e.g., to schedule, plan). Responses were scored on a scale ranging from 0% (*not at all confident*) to 100% (*extremely confident*). Both task (4 items; α’s ≥ .86) and self-regulatory efficacy (14 items; α’s ≥ .90) had strong internal consistency. The overall scale mean was used for all analyses.

### Sample size determination

Please refer to the published protocol for a thorough description of the sample size determination [17]. Briefly, 15 participants per group were needed to detect a significant within-between interaction in cardiorespiratory fitness, our main outcome. This was based on a two-tailed alpha of 0.05 and 80% power, assuming a medium correlation amongst repeated measures of r = 0.5, a pooled mean of 20.8 (*SD* = 4.0) ml/kg/min [15], and a Cohen’s d = .48 difference between HIIT and MICT [2] (calculated using G*Power v3.1). However, our secondary outcome of MVPA typically produces greater measurement variability and we opted to determine the sample size based on the sample size requirements for detecting a clinically relevant within-group change in MVPA. To detect a difference of 10-min average MVPA per day within conditions, assuming a standard deviation of 16 [24], with 80% power at *p <* 0.05, 41 participants per group were required. A conservative ~ 25% loss to follow-up is anticipated and therefore the trial aimed to recruit 50 participants per group (i.e., 100 participants to be randomized).

### Analyses

Linear mixed effect regression was used to assess the change in outcomes at month 3, 6, 9, and 12 (relative to baseline) within and between each treatment group. The regression analysis considered the change in the measurements between the follow-up visits and baseline as the outcome. Time was considered as a categorical variable and an unstructured covariance matrix, which was allowed to differ by treatment group, was used to model the correlation over time. Other than data for self-efficacy and vigorous minutes, which are described below, our data met multivariate assumptions. For self-efficacy, the distribution was non-parametric, so analyses were based on quantile regression to compare the median change in the measurements. For vigorous minutes, due to the distribution of the data, Poisson mixed effects model was used. Analyses were performed using SAS 9.4 (SAS Institute, Cary NC).

## Results

### Demographics

**Fig. 1:**
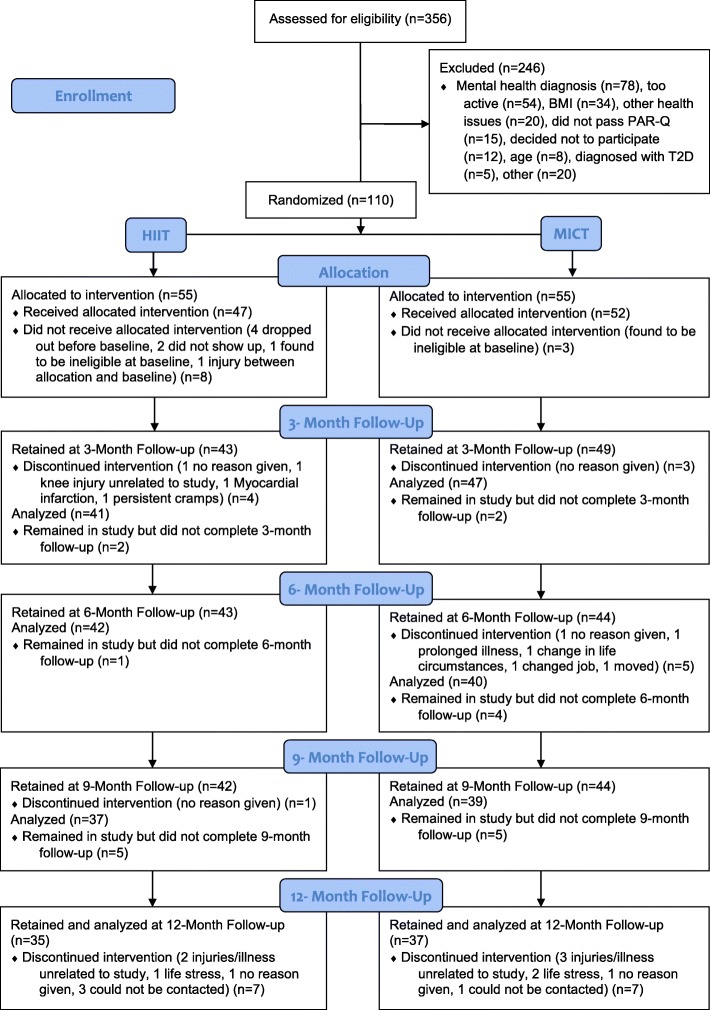
Consort participant flow diagram

Baseline characteristics of the 99 low-active and overweight adults (*M*_*age*_ = 50.9, *SD* = 9.40) randomized to either HIIT (*n* = 47) or MICT (*n* = 52) are reported in Table 1. See Fig. 1 for consort participant flow diagram.

**Table 1:** Descriptive statistics for individuals that took part in the intervention

Variables	All (*N* = 99)	HIIT (*n* = 47)	MICT (*n* = 52)
Age (years)	50.9 (9.40)	51.8 (8.80)	50.0 (9.90)
Gender, *n* (%)			
Male	28 (28.20)	13 (27.70)	15 (28.90)
Female	69 (69.70)	33 (70.20)	36 (69.20)
Did not answer	2 (2.10)	1 (2.10)	1 (1.90)
Body Mass (kg)	89.3 (20.36)	89.4 (21.70)	89.3 (19.30)
Waist circumference (cm)	108.0 (15.10)	108.4 (15.70)	107.6 (14.70)
VO_2_ absolute (L/min)	2.01 (0.63)	2.0 (0.55)	2.02 (0.70)
VO_2_ relative (mL/kg/min)	22.81 (5.75)	22.65 (4.95)	22.95 (6.43)
MVPA 10+	36.33 (51.29)	31.33 (45.77)	40.61 (55.71)
MVPA adherence	0.29 (0.36)	0.33 (0.40)	0.25 (0.32)
Task self-efficacy	82.35 (14.97)	79.47 (15.72)	84.95 (13.88)
Self-regulatory efficacy	71.6 (14.67)	75.63 (14.05)	77.47 (15.29)
Ethnic Origin, *n* (%)			
Caucasian	88 (88.90)	41 (87.20)	47 (90.40)
Latin American	2 (2.02)	1 (2.10)	1 (1.90)
Asian	3 (3.03)	2 (4.30)	1 (1.90)
Native/Aboriginal	2 (2.02)	1 (2.10)	1 (1.90)
Other	2 (2.02)	1 (2.10)	1 (1.90)
Did not answer	2 (2.02)	1 (2.10)	1 (1.90)
Annual Household Income, *n* (%)			
$0 – $24,999	4 (4.04)	1 (2.10)	3 (5.80)
$25,000 - $49,999	11 (11.10)	4 (8.50)	7 (13.50)
$50,000 - $74,999	18 (18.20)	11 (23.40)	7 (13.50)
$75,000 - $ 99,999	20 (20.20)	11 (23.40)	9 (17.30)
$100,000 +	44 (44.40)	19 (40.40)	25 (48.10)
Did not answer	2 (2.02)	1 (2.10)	1 (1.90)
Education, *n* (%)			
High school	13 (13.10)	8 (17.02)	5 (9.60)
College Diploma	32 (32.30)	17 (36.20)	15 (28.80)
Bachelors Degree	35 (35.40)	14 (29.80)	21 (40.40)
Post-Graduate Degree	16 (16.20)	6 (12.80)	10 (19.20)
Did not answer	3 (3.03)	2 (4.30)	1 (1.90)
Marital Status, *n* (%)			
Single	10 (10.10)	5 (10.60)	5 (9.60)
Married	72 (72.70)	37 (78.70)	35 (67.30)
Common-law	5 (5.10)	1 (2.10)	4 (7.70)
Divorced	7 (7.10)	2 (4.30)	5 (9.60)
Widowed	2 (2.00)	1 (2.10)	1 (1.90)
Did not answer	3 (3.03)	1 (2.10)	2 (3.85)

Note: All values are mean (SD) unless indicated as *n* (%). *n* = 2 missing value

The linear mixed effects results examining within- and between-group mean change from baseline to 3-, 6-, 9-, and 12-month post-intervention (with associated 95% confidence interval and *p*-values) for all variables are reported in Table 2. See Additional file 2 for group-level descriptive statistics across the study (i.e., *n*, *M*, *SD*).

**Table 2:** Estimated change relative to baseline (with associated 95% confidence intervals and *p*-values)

		HIIT	*P*-value	MICT	*P*-value	Between group differences	*P*-value
VO_2_ absolute (L/min)	Month 6	0.15 (0.06, 0.24)	0.002	0.12 (0.05, 0.20)	0.001	0.03 (−0.09, 0.14)	0.66
	Month 12	0.16 (0.08, 0.23)	< 0.001	0.10 (0.02, 0.17)	0.014	0.06 (−0.04, 0.17)	0.25
	Average of the 12 months	0.15 (0.08, 0.23)	< 0.001	0.11 (0.05, 0.18)	0.001	0.04 (−0.06, 0.14)	0.38
VO_2_ relative (mL/kg/min)	Month 6	1.79 (0.68, 2.90)	0.002	1.79 (0.75, 2.83)	0.001	0.00 (−1.52, 1.52)	1.00
	Month 12	1.94 (0.88, 2.99)	< 0.001	1.52 (0.50, 2.54)	0.004	0.42 (−1.05, 1.88)	0.57
	Average of the 12 months	1.86 (0.87, 2.85)	< 0.001	1.66 (0.69, 2.62)	0.001	0.21 (−1.17, 1.59)	0.76
Watts peak	Month 6	9.51 (3.83, 15.19)	0.001	8.12 (3.86, 12.38)	< 0.001	1.39 (−5.71, 8.48)	0.70
	Month 12	11.26 (6.53, 15.99)	< 0.001	4.97 (0.25, 9.70)	0.04	6.28 (−0.40, 12.97)	0.07
	Average of the 12 months	10.38 (5.75, 15.02)	< 0.001	6.55 (2.37, 10.73)	0.003	3.83 (−2.40, 10.07)	0.22
MVPA 10+	Month 3	49.39 (30.15, 68.64)	< 0.001	84.03 (53.00, 115.07)	< 0.001	−34.64 (−71.16, 1.88)	0.06
	Month 6	29.36 (9.24, 49.48)	0.004	91.66 (63.91, 119.40)	< 0.001	−62.30 (−96.57, −28.03)	< 0.001
	Month 9	39.80 (16.02, 63.57)	0.001	54.45 (25.92, 82.98)	< 0.001	−14.65 (−51.79, 22.49)	0.44
	Month 12	24.17 (−7.49, 55.83)	0.13	44.26 (19.16, 69.37)	< 0.001	−20.09 (−60.50, 20.31)	0.33
	Average of the 12 months	35.68 (17.22, 54.14)	< 0.001	68.60 (48.66, 88.54)	< 0.001	−32.92 (− 60.09, − 5.75)	0.02
Vigorous PA	Month 3	6.24 (2.09, 18.58)	0.001	2.09 (0.80, 5.42)	0.13	2.99 (0.70, 12.73)	0.14
	Month 6	3.60 (1.21, 10.72)	0.021	3.47 (1.24, 9.72)	0.018	1.04 (0.23, 4.66)	0.96
	Month 9	6.70 (2.19, 20.50)	< 0.001	2.88 (1.05, 7.89)	0.040	2.33 (0.52, 10.51)	0.27
	Month 12	3.35 (1.06, 10.62)	0.040	2.02 (0.69, 5.89)	0.20	1.66 (0.34, 8.01)	0.53
	Average of the 12 months	4.74 (1.93, 11.67)	< 0.001	2.55 (1.16, 5.58)	0.020	1.86 (0.56, 6.15)	0.307
Moderate PA	Month 3	58.54 (34.77, 82.32)	< 0.001	79.52 (39.63, 119.40)	< 0.001	−20.97 (−67.40, 25.46)	0.37
	Month 6	17.13 (−6.67, 40.92)	0.16	93.71 (60.45, 126.97)	< 0.001	−76.58 (− 117.48, −35.69)	< 0.001
	Month 9	19.00 (−6.50, 44.50)	0.14	67.61 (38.51, 96.71)	< 0.001	−48.61 (−87.30, −9.92)	0.014
	Month 12	−7.70 (−41.10, 25.70)	0.65	49.13 (13.38, 84.88)	0.007	−56.83 (− 105.76, − 7.91)	0.023
	Average of the 12 months	21.74 (2.05, 41.44)	0.031	72.49 (48.66, 96.32)	< 0.001	−50.75 (−81.67, − 19.83)	0.001
Moderate-to-vigorous PA	Month 3	68.47 (45.32, 91.63)	< 0.001	86.36 (43.94, 128.78)	< 0.001	−17.88 (−66.21, 30.44)	0.47
	Month 6	24.39 (0.45, 48.33)	0.046	98.98 (65.49, 132.46)	< 0.001	−74.59 (− 115.75, − 33.43)	< 0.001
	Month 9	26.31 (0.03, 52.58)	0.050	72.79 (41.86, 103.71)	< 0.001	−46.48 (−87.06, −5.90)	0.025
	Month 12	−2.24 (−35.82, 31.33)	0.90	61.61 (24.24, 98.99)	0.001	−63.86 (− 114.10, − 13.62)	0.013
	Average of the 12 months	29.23 (9.68, 48.78)	0.004	79.93 (55.44, 104.42)	< 0.001	−50.70 (−82.04, − 19.37)	0.002
Propotion of MVPA prescription achieved	Month 3	0.39 (0.25, 0.52)	< 0.001	0.35 (0.21, 0.48)	< 0.001	0.04 (−0.15, 0.23)	0.69
	Month 6	0.22 (0.08, 0.35)	0.002	0.39 (0.28, 0.50)	< 0.001	−0.18 (− 0.35, − 0.00)	0.05
	Month 9	0.27 (0.09, 0.45)	0.003	0.25 (0.13, 0.36)	< 0.001	0.02 (−0.19, 0.23)	0.83
	Month 12	0.13 (−0.06, 0.32)	0.19	0.19 (0.08, 0.29)	< 0.001	−0.06 (− 0.28, 0.16)	0.61
	Average of the 12 months	0.25 (0.11, 0.39)	< 0.001	0.29 (0.21, 0.38)	< 0.001	−0.04 (− 0.20, 0.12)	0.59
Weight (kg)	Month 6	−0.26 (−1.10, 0.58)	0.54	−1.28 (−3.32, 0.77)	0.22	1.02 (−1.19, 3.23)	0.36
	Month 12	0.00 (−1.32, 1.31)	1.00	−1.48 (−3.92, 0.97)	0.23	1.48 (−1.30, 4.25)	0.29
	Average of the 12 months	−0.13 (−1.09, 0.82)	0.78	−1.38 (−3.57, 0.81)	0.21	1.25 (−1.14, 3.64)	0.30
Waist circumference (cm)	Month 6	−1.89 (−3.14, −0.65)	0.003	−3.62 (−5.84, − 1.39)	0.002	1.72 (− 0.82, 4.27)	0.18
	Month 12	−2.62 (−4.24, − 1.01)	0.002	−4.95 (−7.41, − 2.49)	< 0.001	2.32 (− 0.62, 5.26)	0.12
	Average of the 12 months	− 2.26 (− 3.55, − 0.97)	< 0.001	−4.28 (−6.54, − 2.02)	< 0.001	2.02 (− 0.58, 4.63)	0.13
Body fat (%)	Month 6	− 0.98 (− 1.55, − 0.41)	0.001	−0.93 (− 1.80, − 0.07)	0.04	−0.05 (− 1.09, 0.99)	0.93
	Month 12	−1.68 (− 2.47, − 0.90)	< 0.001	−1.90 (− 2.88, − 0.92)	< 0.001	0.22 (− 1.04, 1.47)	0.73
	Average of the 12 months	− 1.33 (− 1.94, − 0.73)	< 0.001	− 1.42 (− 2.27, − 0.56)	0.002	0.08 (− 0.96, 1.13)	0.87
Task self-efficacy	Month 6	12.50 (6.60, 18.40)	< 0.001	7.50 (3.07, 11.93)	0.001	5.00 (−2.38, 12.38)	0.18
	Month 12	10.00 (2.87, 17.13)	0.007	5.00 (−2.81, 12.81)	0.21	5.00 (−5.58, 15.58)	0.35
Self-regulatory efficacy	Month 6	1.43 (− 4.62, 7.47)	0.64	3.57 (−1.45, 8.59)	0.16	−2.14 (− 10.00, 5.72)	0.59
	Month 12	−1.43 (− 12.39, 9.53)	0.80	2.86 (− 4.08, 9.80)	0.42	−4.29 (− 17.26, 8.69)	0.51

All values are estimated change (95% CI)

Number of participants in HIIT: 3-months (*n* = 43); 6-months (*n* = 42); 9-months (*n* = 37); 12-months (*n* = 35)

Number of participants in MICT: 3-months (*n* = 49); 6-months (*n* = 44); 9-months (*n* = 39); 12 months (*n* = 37)

### Cardiorespiratory fitness

**Fig. 2:**
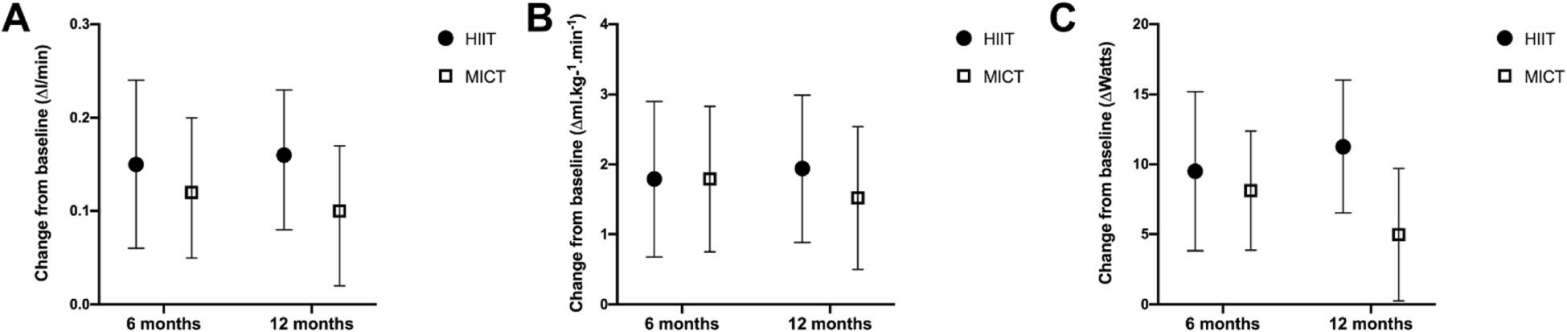
**a**-**c** Mean change in cardiorespiratory fitness from baseline to 6- and 12-months post-intervention in HIIT and MICT groups. HIIT = High-Intensity Interval Training. MICT = Moderate-Intensity Continuous Training. Cardiorespiratory fitness was assessed by a continuous incremental ramp maximal exercise test on an electronically braked cycle ergometer. **a** presents changes in absolute VO2peak. **b** presents changes in relative VO2peak. **c** presents changes in peak power output

Figures 2a-c graphically depict changes in CRF (absolute VO_2peak_ relative VO_2peak_, and W_peak_) from baseline to 6- and 12-months post-intervention. Compared to baseline, both groups significantly improved on all three measures of CRF at 6-months, 12-months, and average of both timepoints. There were no significant between-group differences on any CRF measures.

### Purposeful MVPA

Both groups significantly increased minutes of MVPA10+ from baseline across the 12 months following the intervention (average of the 3-, 6-, 9-, and 12-month timepoints). There was a significant between-group difference across the year of free-living activity post-intervention as those randomized to MICT engaged in a significantly greater amount of MVPA10+ compared to those randomized to HIIT. Within groups, there were significant increases in MVPA10+ above baseline at each individual timepoint (3, 6, 9, and 12-months) except at the 12-month timepoint for HIIT, which failed to reach statistical significance (*p* = 0.13). Table 2 contains a breakdown of the changes in minutes of vigorous PA, moderate PA, and moderate-to-vigorous PA. Those in HIIT and MICT also reported similar within-groups changes in the proportion of MVPA prescription achieved throughout the 1 year follow-up.

### App-based self-monitored exercise

Those randomized to the MICT condition logged MICT an average of 3.8x/week (*SD* = 1.2) in the first 6 months following the intervention and 3.3x/week (*SD* = 1.3) in the second 6 months. Those randomized to the HIIT condition logged HIIT an average of 1.9x/week (*SD* = 0.9) in the first 6 months and 1.0x/week (*SD* = 0.9) in the second 6 months. Interestingly, those in HIIT also reported engaging in MICT an average of 1.2x/week (*SD* = 1.0; 3.1x/week total exercise) in the first 6 months and 1.4x/week (*SD* = 1.2; 2.4x/week of total exercise) in the second 6 months.

### Anthropometrics

Compared to baseline, both groups significantly reduced their waist circumference and total body fat percentage at 6-months, 12-months, and the average of both time points with no significant between-group differences. There were no significant within- or between-group changes in body mass observed throughout the study.

### Self-efficacy

There were no significant within- or between-group changes in self-regulatory efficacy observed throughout the study. Those in HIIT significantly increased their task self-efficacy at 6- and 12-months, while those in MICT significantly increased their task self-efficacy at 6-months but not at 12-months. There were no significant between-group differences at either 6- or 12-months in self-regulatory or task self-efficacy.

## Discussion

The main objective of this randomized trial was to compare CRF and accelerometer-assessed free-living physical activity over the 12 months following a brief 2-week behaviour change counselling intervention that included HIIT or standard care MICT. Individuals who were previously low-active and with overweight or obesity were able to sustain improvements in fitness across 1 year in both conditions. While both conditions increased physical activity above baseline, individuals randomized to a prescription of 150 min per week of MICT engaged in significantly more accelerometer-measured physical activity than individuals who were prescribed 75 min per week of HIIT. Cardiometabolic risk factors including waist circumference and body fat percentage were also improved in both conditions throughout the 12 months following the intervention. Overall, these findings suggest a brief behaviour change counselling intervention, coupled with either HIIT or MICT, promotes improvements in CRF, exercise, and body composition over 12 months in free-living conditions.

### Free-living changes in cardiorespiratory fitness and adherence to HIIT and MICT

There are a limited number of trials examining free-living adherence to HIIT as compared to MICT. Roy et al. [29] recently reported 12-month free-living adherence to HIIT using a non-randomized study design in which individuals could self-select whether they would engage in HIIT or MICT after completing one supervised bout of each exercise modality. In that study, ~ 20% of individuals who *self-selected* to engage in HIIT over traditional MICT were still engaging in HIIT 1 year later. Unfortunately, incongruent measures of adherence precluded comparisons on rates of adherence between self-selected HIIT and MICT in that recent study [29]. Further, cardiorespiratory fitness was not directly assessed, but the authors did report estimated VO_2peak_ was unchanged from baseline in both HIIT or MICT groups [29]. Our findings demonstrated participants in both groups engaged in ~ 25–30% of the prescribed number of exercise minutes per week (as assessed by accelerometer), with no difference between conditions. Distinct from Roy and colleagues, we demonstrated significant and sustained improvements in absolute and relative VO_2_peak at the group level in both conditions, suggesting both HIIT and MICT are effective for improving CRF across 12 months of free-living conditions when previously low-active individuals receive an evidence-based behaviour change counselling intervention designed to promote exercise engagement.

Total minutes of purposeful exercise (MVPA10+) across 12 months were increased from baseline in both groups, but the increase for MICT was approximately 30 min greater than for HIIT. This is not surprising as the prescription for MICT involved 150 min per week whereas the prescription for HIIT totaled only 75 min per week. Despite engaging in less overall purposeful exercise, those randomized to HIIT had similar improvements in CRF. These findings support the time-efficiency of HIIT for promoting fitness improvements in the real-world under free-living conditions. These improvements are clinically meaningful when interpreted in light of evidence that a 1 ml/kg/min increment in VO_2peak_ is associated with a 10% reduction in cardiovascular mortality risk [30, 31]. Longer-term studies will be needed to determine if greater increases in MVPA10+ in MICT (if sustained) would translate to differences in cardiometabolic health outcomes over time.

Interestingly, app-based self-reported exercise adherence indicated individuals randomized to (and prescribed) HIIT appeared to engage in both HIIT and MICT throughout the 12 months of follow-up. This provides evidence that, despite being prescribed HIIT 3 days per week, the previously inactive individuals in this trial chose to perform a combination of HIIT and MICT. These self-report data highlight potential limitations in accelerometry for determining adherence to HIIT in the real-world, indicate physiological adaptations in the HIIT group at 12 months may not be exclusively attributable to performing HIIT, and suggest prescribing exclusive HIIT may not be optimal for increasing exercise engagement.

### Improvements in body composition

A recent meta-analysis of supervised trials reported interval training (including low-volume HIIT) may be superior to MICT for improving fat loss [3]. Our findings add to the literature by showing both HIIT and MICT lead to reductions in DXA-assessed body fat percentage and waist circumference over 12 months of free-living exercise. One potential reason why we did not see significantly greater fat loss with HIIT could be related to the greater volume of purposeful exercise in those randomized to MICT. A second reason could be a diluted effect size compared to supervised studies given that not all participants were fully adherent to either HIIT or MICT. However, these findings should be interpreted with some caution given diet tends to be the primary driver of weight loss and we did not assess diet in the present study. Coupled with the finding of no change in body mass, these results suggest both HIIT and MICT were effective for improving body composition but not necessarily weight loss. Lifestyle interventions aiming to promote weight loss likely need to incorporate dietary changes in addition to exercise [32, 33].

### Changes in self-efficacy

Individuals will ultimately fail to initiate a new behaviour if they are not confident to perform that behaviour [14]. This may especially be the case for low-active individuals who may not have been successful in the past at performing and sticking to regular exercise. Bolstering self-efficacy throughout the supervised program was necessary to ensure our low-active participants had the confidence to perform and manage free-living exercise following the supervised phase. The observed increases in task self-efficacy suggests behaviour change counselling was effective at enhancing participants’ self-efficacy to perform HIIT or MICT. While we observed modest exercise adherence rates, neither group significantly increased their self-regulatory self-efficacy. This finding was contrary to our a priori expectation, and as such it remains to be ascertained exactly why particpants in both conditions reported improvements in task self-efficacy, but not self-regulatory efficacy.

### Strengths and limitations

The decision to use two active trial arms rather than a no-treatment comparator group was informed by the research question comparing fitness between HIIT and MICT after 1 year of free-living physical activity. While this precluded us from parsing out effects of simply enrolling in a lifestyle intervention, it allowed us to examine the relative effects of free-living HIIT as compared to the traditionally-prescribed MICT. Offering participants the opportunity to self-select the exercise format may have limited the ability to estimate and measure the intensity level of the different types of exercise (e.g., cycling, running). However, this was necessary to be able to provide participants with the opportunity to self-select exercise modality. Self-selection develops autonomy and allowed participants to exercise in the format they would continue to use over the following year in free-living conditions.

We used MVPA10+ as an accelerometer-based measure of exercise because it can more appropriately capture purposeful exercise in the real-world. We have previously shown walking-based HIIT can be identified by accelerometry [11] but accelerometers do have limitations for quantifying HIIT in free-living conditions (e.g., individual vs. standard cutpoints for determining vigorous intensity). While the study was limited by the 28% dropout rate, data were examined using linear mixed models which incorporate baseline and trajectories of missing participants based on a ‘missing at random’ assumption. We assessed participants’ proportion of MVPA prescription achieved. The assessment was limited in that it included purposeful moderate and vigorous exercise, not just the condition-specific intensity. There may have been a drift in exercise intensity within the two groups throughout the one-year of free-living exercise. This assessment helped to credit participants for the purposeful exercise they did perform even though the operationalization did not perfectly align with the prescription.

Physical activity was the only health behaviour targeted in this intervention. Changes in diet were not assessed, and as such, the interpretation of our weight loss findings may be limited given diet, and not physical activity, tends to be the primary driver of weight loss. However, our randomization protocol helps to give confidence that potentially confounding factors, such as dietary changes, were randomly distributed between groups.

## Conclusion

Findings support the notion that 2 weeks of HIIT or MICT combined with behavior change counselling can lead to increased fitness levels for at least 1 year. Our previous pilot research demonstrated the feasibility of six-month adherence to HIIT, in line with the current results. However, over 12 months in this trial, those randomized to MICT achieved significantly more minutes of purposeful physical activity. Greater purposeful physical acitivty in MICT, in which the prescription called for twice as many minutes of exercise (150 vs. 75), points to efficacy of the brief behaviour change counselling protocol in promoting lasting improvements in MVPA. The self-report exercise adherence data also indicated individuals randomized to HIIT also selected to engage in purposeful MICT during 12 months of free-living follow-up, which suggests HIIT may have “spillover” effects to increase other types of exercise and exclusive prescription of HIIT may not be ideal in the real-world. The approach of combining physical acivity training (of any type) with brief evidence-based behaviour change counselling should be explored in future studies in order to enhance adherence post-trial.

## Supplementary information

**Additional file 1.** Consort checklist.

**Additional file 2.** Group-level descriptive statistics for all study outcomes.
