# Supplementary material for: Cardiorespiratory fitness and accelerometer-determined physical activity following one year of free-living high-intensity interval training and moderate-intensity continuous training: a randomized trial
Source: Int J Behav Nutr Phys Act. 2020 Feb 26;17:25. doi: 10.1186/s12966-020-00933-8 (PMC7045584; doi:10.1186/s12966-020-00933-8)
Supplement: Supplementary file 2 — Additional file 2. Group-level descriptive statistics for all study outcomes. [file 12966_2020_933_MOESM2_ESM.pdf]

Supplementary File 1. Group-level descriptive statistics for all study outcomes.

		HIIT	MICT
**VO_2 absolute (L/min)**	**Pre**	2.00 (0.55)	2.02 (0.70)
	**Post**	2.15 (0.57)	2.05 (0.59)
	**Month 6**	2.12 (0.55)	2.05 (0.71)
	**Month 12**	2.08 (0.57)	2.03 (0.56)
**VO_2 relative (mL/kg/min)**	**Pre**	22.65 (4.95)	22.95 (6.44)
	**Post**	24.19 (4.63)	23.43 (5.78)
	**Month 6**	24.61 (6.03)	24.17 (6.60)
	**Month 12**	25.60 (6.38)	24.18 (5.82)
**Watts peak**	**Pre**	170.62 (33.72)	173.12 (47.37)
	**Post**	180.04 (35.80)	176.90 (42.82)
	**Month 6**	177.95 (36.55)	178.20 (53.12)
	**Month 12**	177.67 (36.89)	174.64 (39.51)
**MVPA 10+**	**Pre**	31.33 (45.77)	40.61 (55.71)
	**Month 3**	81.14 (62.23)	127.88 (100.80)
	**Month 6**	62.66 (64.55)	141.70 (93.49)
	**Month 9**	72.66 (66.17)	113.79 (100.07)
	**Month 12**	65.14 (78.99)	97.23 (99.27)
**MVPA adherence**	**Pre**	0.33 (0.40)	0.25 (0.32)
	**Month 3**	0.73 (0.35)	0.62 (0.40)
	**Month 6**	0.57 (0.39)	0.70 (0.33)
	**Month 9**	0.65 (0.39)	0.58 (0.41)
	**Month 12**	0.54 (0.40)	0.49 (0.42)
**Weight (kg)**	**Pre**	89.40 (21.67)	89.26 (19.32)
	**Post**	89.08 (21.11)	88.34 (18.72)
	**Month 6**	87.26 (21.15)	86.15 (16.81)
	**Month 12**	81.46 (13.66)	85.60 (17.34)
**Waist circumference (cm)**	**Pre**	108.41 (15.67)	107.63 (14.72)
	**Post**	107.09 (15.96)	105.71 (14.09)
	**Month 6**	105.41 (15.66)	102.84 (12.99)
	**Month 12**	100.16 (11.23)	100.98 (13.22)
**Body fat (%)**	**Pre**	33.11 (6.63)	33.56 (7.69)
	**Post**	32.63 (6.90)	33.41 (7.68)
	**Month 6**	32.15 (6.56)	33.28 (7.41)
	**Month 12**	30.46 (6.78)	31.78 (7.41)
**Task self-efficacy**	**Pre**	79.47 (15.72)	84.95 (13.88)
	**Post**	98.83 (2.49)	97.03 (6.07)
	**Month 6**	92.44 (18.74)	88.49 (19.87)
	**Month 12**	86.83 (27.07)	89.46 (13.82)
**Self-regulatory efficacy**	**Pre**	75.64 (14.05)	77.47 (15.29)
	**Post**	88.34 (9.71)	91.33 (11.50)
	**Month 6**	76.09 (17.17)	82.42 (17.07)
	**Month 12**	72.53 (21.75)	85.37 (13.64)

Number of participants in HIIT: Pre (n = 47); Post (n = 47); *3*-months (n = 43); 6-months (n = 42); 9-months (n = 37); 12-months (n = 35).
Number of participants in MICT: Pre (n = 52); Post (n = 51); 3-months (n = 49); 6-months (n = 44); 9-months (n = 39); 12-months (n = 37).
